# Landscape of semi-extractable RNAs across five human cell lines

**DOI:** 10.1093/nar/gkad567

**Published:** 2023-07-19

**Authors:** Chao Zeng, Takeshi Chujo, Tetsuro Hirose, Michiaki Hamada

**Affiliations:** Faculty of Science and Engineering, Waseda University, Tokyo 1698555, Japan; Faculty of Life Sciences, Kumamoto University, Kumamoto 8608556, Japan; Graduate School of Frontier Biosciences, Osaka University, Suita 5650871, Japan; Institute for Open and Transdisciplinary Research Initiatives (OTRI), Osaka University, Suita 5650871, Japan; Faculty of Science and Engineering, Waseda University, Tokyo 1698555, Japan; AIST-Waseda University Computational Bio Big-Data Open Innovation Laboratory (CBBD-OIL), National Institute of Advanced Industrial Science and Technology, Tokyo 1698555, Japan; Graduate School of Medicine, Nippon Medical School, Tokyo 1138602, Japan

## Abstract

Phase-separated membraneless organelles often contain RNAs that exhibit unusual semi-extractability using the conventional RNA extraction method, and can be efficiently retrieved by needle shearing or heating during RNA extraction. Semi-extractable RNAs are promising resources for understanding RNA-centric phase separation. However, limited assessments have been performed to systematically identify and characterize semi-extractable RNAs. In this study, 1074 semi-extractable RNAs, including ASAP1, DANT2, EXT1, FTX, IGF1R, LIMS1, NEAT1, PHF21A, PVT1, SCMH1, STRG.3024.1, TBL1X, TCF7L2, TVP23C-CDRT4, UBE2E2, ZCCHC7, ZFAND3 and ZSWIM6, which exhibited consistent semi-extractability were identified across five human cell lines. By integrating publicly available datasets, we found that semi-extractable RNAs tend to be distributed in the nuclear compartments but are dissociated from the chromatin. Long and repeat-containing semi-extractable RNAs act as hubs to provide global RNA–RNA interactions. Semi-extractable RNAs were divided into four groups based on their k-mer content. The NEAT1 group preferred to interact with paraspeckle proteins, such as FUS and NONO, implying that RNAs in this group are potential candidates of architectural RNAs that constitute nuclear bodies.

## Graphical Abstract

**Graphical Abstract:**
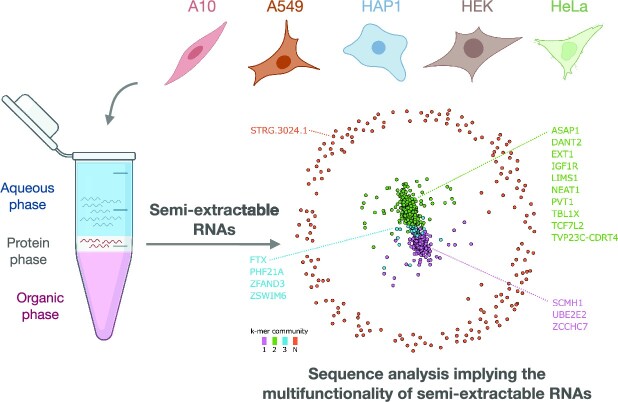

## INTRODUCTION

Liquid–liquid phase separation (LLPS) is a biological phenomenon in which macromolecules, such as proteins or nucleic acids, are spatially organized into membrane-less organelles (MLOs; also called biomolecular condensates) (1). MLOs usually maintain their stable structures through multivalent interactions of molecules that act in diverse biological processes ranging from macromolecular biogenesis to gene regulation (2–4). MLOs are highly dynamic structures, whose components rapidly exchange between other condensates and the surrounding milieu (5–9), implying that MLOs are sensitive to internal and external signals. LLPS provides a new framework for our understanding of human health and disease (10–12). Phase-separated MLOs that have been discovered and studied include the nucleolus, paraspeckle, nuclear speckle, Cajal body, PML nuclear body, P-body, stress granule, germ granule and mRNP granule (3). The role of proteins in LLPS and their regulation has been the focus of attention (1,13–15). However, based on accumulating evidence, RNAs, especially long non-coding RNAs (lncRNAs), play a crucial role in the process of phase separation (16–21).

As a remarkable example, nuclear paraspeckle assembly transcript 1 (NEAT1) is an architectural lncRNA that mediates the assembly of paraspeckles by driving phase separation (22–25). Two major isoforms are generated from the NEAT1 gene locus, and the longer isoform NEAT1_2 serves as a molecular scaffold for the formation of RNA–protein and RNA–RNA interactions (19,26). Paraspeckles form a core–shell spheroidal structure, in which the shell contains the 5′ and 3′ regions of NEAT1_2 and some specific proteins, whereas the core consists of the middle region of NEAT1_2 and Drosophila behavior/human splicing (DBHS) proteins (27). According to further studies, the NEAT1_2 middle region contains redundant subdomains that sequester RNA-binding proteins (RBPs), such as non-POU domain-containing octamer-binding protein (NONO) and splicing factor proline and glutamine rich (SFPQ), to initiate paraspeckle assembly (28). Note that both NONO and SFPQ are members of the DBHS family of proteins. Interestingly, when a conventional RNA extraction method using AGPC (acid guanidinium thiocyanate–phenol–chloroform) reagent such as TRIzol is employed, most of the NEAT1 is retained in the protein layer between the aqueous phase and organic phase, resulting in a low extraction level. However, after the phase-separated structures are disrupted by an improved RNA extraction through needle shearing or heating, NEAT1 is released into the aqueous solution, and its extraction level can be 20-fold higher than that obtained via the conventional method. Such a property of NEAT1 is termed ‘semi-extractability’ (29). The semi-extractability of NEAT1 strongly depended on the prion-like domain of a paraspeckle RBP, FUS, implying that extensive multivalent interactions may cause semi-extractability (22). In addition to NEAT1, several other newly detected semi-extractable RNAs were observed to form granule-like foci in a previous study (29). Accordingly, RNAs in the phase-separated structures may commonly possess semi-extractability owing to multivalent forces. The systematic identification and characterization of semi-extractable RNAs could aid in the discovery of RNAs associated with phase-separated MLOs and provide insights into LLPS biology.

In this study, we developed a bioinformatic pipeline to define 1074 semi-extractable RNAs for the first time in five human cell lines. As controls, we also identified 6695 extractable RNAs from all five cell lines. Extractable RNAs were defined as RNAs without pronounced expression changes using the improved RNA extraction method, indicating that they were recovered well by the conventional method. Compared with extractable RNAs, semi-extractable RNAs prefer to be transcribed from repressed and repetitive/heterochromatin regions that are clustered in the nuclear compartments. Long and AU-rich semi-extractable RNAs contain more repetitive sequences than expected and interact frequently with other RNAs. Semi-extractable RNAs can be broadly classified into four different groups based on their sequence composition, with the semi-extractable RNAs of the NEAT1 group preferring to bind paraspeckle RBPs (e.g. NONO and FUS), suggesting their potential role as architectural RNAs.

## MATERIALS AND METHODS

### Cellular RNA extraction and sequencing

A10, A549, HEK293 and HeLa cells were grown in Dulbecco’s modified Eagle’s medium (DMEM; GIBCO) supplemented with 10% fetal bovine serum (FBS) in a humidified, 37°C atmosphere with 5% CO_2_. HAP1 cells were grown similarly in Iscove's modified Dulbecco's medium (IMDM; GIBCO) supplemented with 10% FBS. RNA was extracted essentially as previously described (29) with some modifications as described below. TRI reagent (MRC) was added to cells at a ratio of 1 ml of TRI reagent to 1 × 10^7^ cells. Subsequently, for conventional RNA extraction, cell lysates in TRI reagent were extracted according to the manufacturer’s instructions. For improved RNA extraction, cell lysates in TRI reagent were diluted to 1 × 10^6^ cells/ml using TRI reagent and passed 100 times through a 20 gauge needle. Subsequently, total RNA was extracted according to the manufacturer’s instructions. An aliquot of 1 μg of purified RNA was subjected to rRNA depletion using a Ribo-Zero Gold kit (Epicentre). Sequencing libraries were constructed from 100 ng of total RNA using a Truseq stranded mRNA Library Prep kit (Illumina) without the poly(A) selection step. Subsequently, sequencing was performed using a Hiseq3000 (Illumina) with the 36 bp single-end or 101 bp paired-end method.

### RNA-seq analysis

Paired-end reads were trimmed using cutadapt (v3.5) (30) with the following parameters: -a AGATCGGAAGAGCACACGTCTGAACTCCAGTCAC -A AGATCGGAAGAGCGTCGTGTAGGGAAAGAGTGTA --overlap 5 --trim-n --max-n 1 --minimum-length 50:50. For single-end reads, the adapter removal step was skipped. The human genome sequence (hg38) and gene annotation were downloaded from the GENCODE (v43) project (31). Abundant RNAs were extracted from the gene annotation according to the criteria of transcript length <200 nt or transcript biotype containing tRNA, microRNA (miRNA), small nucleolar RNA (snoRNA) and rRNA. First, the reads were mapped to the abundant RNAs using STAR (v2.7.10a) (32) when multi-mapped reads were allowed. Thereafter, the unmapped reads were mapped to the genome using STAR with the following parameter: --outFilterMultimapNmax 1. Transcript/exon/intron FPKM (fragments per kilobase of exon per million mapped reads) was estimated using the StringTie (v2.2.1) (33) quantification mode (-e) with default parameters.

The reference transcriptome for quantification (FPKM and read count) was constructed in the following steps. For each gene, all annotated exons were collapsed and merged to a unique set of exons that constitutes a single transcript (hereinafter referred to as ‘representative transcript’). RNA-seq, obtained using the improved RNA extraction method, was used to assess intron retention and to assemble novel transcripts from intergenic regions. To assess the intron retention for a representative transcript, the retention score (RS) for the *i*-th intron was calculated as follows:


(1) \begin{eqnarray*} RS_i &= \frac{2*\sum I_{i}^{j}}{\sum E_{i}^{j} + \sum E_{i+1}^{j}}, \end{eqnarray*}

where *E* and *I* indicate the FPKM of the exon and intron, respectively. *j* ∈{A10, A549, HAP1, HEK, HeLa}. We defined a retained intron as its FPKM > 0.1 and RS > 0.1. All retained introns in a representative transcript were merged with it to form another one that we refer to as an ‘intron-retaining transcript’. Note that a gene may hold both a representative transcript and an intron-retaining transcript in the reference transcriptome. For each sample, StringTie assembles transcripts based on mapped reads with the parameter: --rf. The transcripts obtained from all samples were grouped by forward and reverse strands and then merged separately using StringTie merge mode (--merge) with default parameters. Finally, the two groups of transcripts were concatenated into the reference transcriptome. After removal of those transcripts that overlap with the representative transcripts and their 5000 nt upstream and downstream regions, we obtained ‘intergenic transcripts’. The above-mentioned representative transcripts, intron-retaining transcripts and intergenic transcripts were combined into the complete reference transcriptome.

For the differential expression analysis between conditions (the conventional RNA extraction and the improved extraction), the transcript-level count of mapped reads was estimated using featureCounts (v2.0.1) (34) with the following parameters: -O -s 2. Then, the fold change (FC) and the *P*-value of the transcript were calculated using edgeR (v3.28.1) (35). To apply edgeR analysis on the partial datasets without biological replicates, we derived an average dispersion of 0.125 from the other datasets as an empirical parameter. For each cell line, transcripts were categorized based on the following criteria: those with average FPKM < 0.5 were labeled as low expressed (Low); those with average FPKM ≥ 0.5, *P*-value < 0.05 and log_2_FC > 0 were categorized as up-regulated (Up); those with average FPKM ≥ 0.5, *P*-value < 0.05 and log_2_FC < 0 were categorized as down-regulated (Down); and the remaining transcripts were considered non-significant (NS). We consider that a transcript categorized as ‘Up’ in a cell line indicates that it exhibits semi-extractability for the cell line. Transcripts can be labeled ‘common’, ‘specific’ or ‘switch’ based on their categorization in A10, A549, HAP1, HEK and HeLa cell lines. If a transcript is categorized as one category in all five cell lines, it is labeled ‘common’ as either ‘Up common’, ‘Down common’ or ‘Low common’. If a transcript is categorized as two categories in all five cell lines, with one being a Low category, it is labeled ‘specific’. Transcripts that do not fall into the above categories are labeled as ‘switch’, e.g. ‘Up–NS switch’ or ‘Down–NS switch’. The label ‘Up common’ indicates that the transcript is consistently semi-extractable in all five cell lines, while ‘Up specific’ means it is specifically expressed in some cell lines and is semi-extractable in all of them. For subsequent meta-analysis, we defined transcripts labeled as ‘Up common’ or ‘Up specific’ as a set of semi-extractable RNAs (denoted as ‘SE’), and transcripts labeled as ‘NS common’ as a set of extractable RNAs (denoted as ‘EX’). Note that in SE or EX, the representative transcript is removed when both the representative transcript and the intron-retaining transcript of a gene are present. Additionally, the reference transcriptome (including representative transcripts, intron-retaining transcripts and intergenic transcripts) were prepared as background controls (denoted as ‘BG’).

The UCSC genome browser (36) was used to visualize the reference transcriptome and read coverage. To visualize read coverage, the mapped reads in BAM format were indexed using Samtools (v1.14) (37) and then converted to bigWig format with bamCoverage (v3.5.1) (38) using the following parameters: --filterRNAstrand forward/reverse --scaleFactor 1/-1 -bs 1 --normalizeUsing RPKM.

### Chromatin state

The chromatin states of HeLa cells were downloaded from the ENCODE (39) project (http://hgdownload.cse.ucsc.edu/goldenpath/hg19/encodeDCC/wgEncodeAwgSegmentation/wgEncodeAwgSegmentationChromhmmHelas3.bed.gz). These chromatin states were predicted using a trained ChromHMM (40) model based on multiple chromatin datasets, including ChIP-seq data for various histone modifications. The annotations of chromatin states that were on hg19 were remapped to hg38 using the pyliftover package (https://github.com/konstantint/pyliftover). The chromatin state prefixes were re-annotated as follows: (i) Active Promoter: Tss and TssF; (ii) Promoter Flanking: PromF; (iii) Inactive Promoter: PromP; (iv) Candidate Strong enhancer: Enh and EnhF; (v) Candidate Weak enhancer/DNase: EnhWF, EnhW, DNaseU, DNaseD; (vi) Distal CTCF/Candidate Insulator: CtrcfO and Ctcf; (vii) Transcription associated: Gen5′, Elon, ElonW, Gen3′, Pol2, H4K20. (viii) Low activity proximal to active states: Low. (ix) Polycomb repressed: ReprD, Repr and ReprW; and (x) Heterochromatin/Repetitive/Copy Number Variation: Quies, Art. The chromatin states were then intersected with semi-extractable and extractable RNAs using the BEDTools (41) intersect command.

### Subcellular localization

APEX-seq data for HEK cells were obtained from GSE116008. APEX-seq is an RNA sequencing method coupled with direct RNA proximity labeling (42). For each cell compartment, we measured the enrichment of a transcript in that compartment (termed subcellular localization) by calculating the FC in the abundance of that transcript between labeled and unlabeled libraries. Accordingly, the RNA-seq reads were first subjected to adapter trimming using Trimmomatic (v0.39) (43) with the following parameters: ILLUMINACLIP:adapter.fa:2:30:4 TRAILING:20 MINLEN:36. Then the reads were uniquely mapped to the human genome using STAR, and the transcript abundance was estimated using StringTie. Finally, subcellular localization (log_2_FC in transcript abundance) was calculated using an in-house script. For a transcript, a higher value of a subcellular localization value indicates a higher enrichment in the corresponding cell compartment.

### Minimum free energy analysis

Using a transcript, subsequences of 300 nt length were extracted from its 5′ and 3′ ends. Transcripts <600 nt in length were removed beforehand. These subsequences were subjected to minimum free energy (MFE) calculations using RNAfold (v2.5.0) (44) with default parameters. Generally, a lower MFE value indicates a more stable RNA structure.

### RNA–chromatin interaction

*In situ* mapping of RNA-Genome Interactome (iMARGI) data of HEK cells were downloaded from GSM3478205. iMARGI is a DNA sequencing method based on RNA–DNA proximity ligation *in situ* inside an intact nucleus (45). The genomic coordinates of the RNA ends in the RNA–DNA interactions were extracted from the processed iMARGI data. The RNA ends were then intersected with transcripts using the BEDTools intersect command. To measure the extent to which a transcript interacts with chromatin, the fraction of transcript regions covered by iMARGI RNA ends was calculated. This fraction ranged from 0 to 1, with a higher fraction suggesting a more frequent interaction between the transcript and chromatin.

### RNA–RNA interaction

RNA interaction hubs (termed ‘hub RNAs’) were derived from RIC-seq (RNA *in situ* conformation sequencing) (46) and PARIS (psoralen analysis of RNA interactions and structures) (47). Hub RNAs exhibited stronger *trans*-interactions than other RNAs. RIC-seq can detect protein-mediated RNA–RNA interactions, while PARIS can directly identify RNA duplexes *in trans* across the transcriptome. The hub RNAs defined by RIC-seq were obtained from a previous study (46). Hub RNAs defined by PARIS were downloaded from the RISE database (48) and reserved for RNAs associated with >20 RNAs simultaneously in HeLa cells. Only 9712 expressed genes (average FPKM ≥ 0.5, removal of intergenic genes and genes without gene names) in HeLa cells were considered when computing the enrichment of overlap.

### Repeat density

The genomic coordinates of the repeat sequences were extracted using RepeatMasker (hg38, repeat library 20140131; https://www.repeatmasker.org/species/hg.html). For a transcript, BEDtools was used for intersection with repeat sequences. The fraction of the transcript that overlapped with repeat sequences, termed repeat density, was then calculated using an in-house script.

### Sequence motif analysis

Human RBP-binding sequence motifs (position weight matrix format) were downloaded from the CISBP-RNA database (http://cisbp-rna.ccbr.utoronto.ca; accessed on March 12, 2022). For a transcript, FIMO (v5.4.1) (49) scanned RBP-binding sites based on the above motifs using the following parameters: --norc --thresh 0.01 --motif-pseudo 0.1 --max-stored-scores 100 000 000. Given a transcript, the binding score of a certain RBP was defined as the number of binding sites of this RBP normalized by the transcript length. The binding preference of an RBP for semi-extractable RNAs is the ratio (log_2_ scale) of its average binding score to that of extractable RNAs.

### k-mer analysis

Semi-extractable RNAs were functionally classified using the k-mer content-based SEEKR (50) algorithm. First, seekr_kmer_counts was used to count the frequency of k-mer occurrence with the following parameter: -k 6. Thereafter, seekr_pearson was used to calculate the similarity matrix. Finally, seekr_graph segmented the RNA sequences into different communities based on the similarity matrix described above and the following parameters: 0.19 --louvain. The network graph of the semi-extractable RNAs was visualized using Gephi (v0.9) (51) with a Yifan Hu proportional layout.

### Gene Ontology analysis

Gene Ontology (GO) analysis of the semi-extractable genes across the five cell lines was conducted using g:Profiler (version: e105_eg52_p16_e84549f) (52). Statistical domain scope: only annotated genes; significance threshold: Bonferroni correction; user threshold: 0.001.

## RESULTS

### Reference transcriptome for identifying semi-extractable RNAs

**Figure 1.**
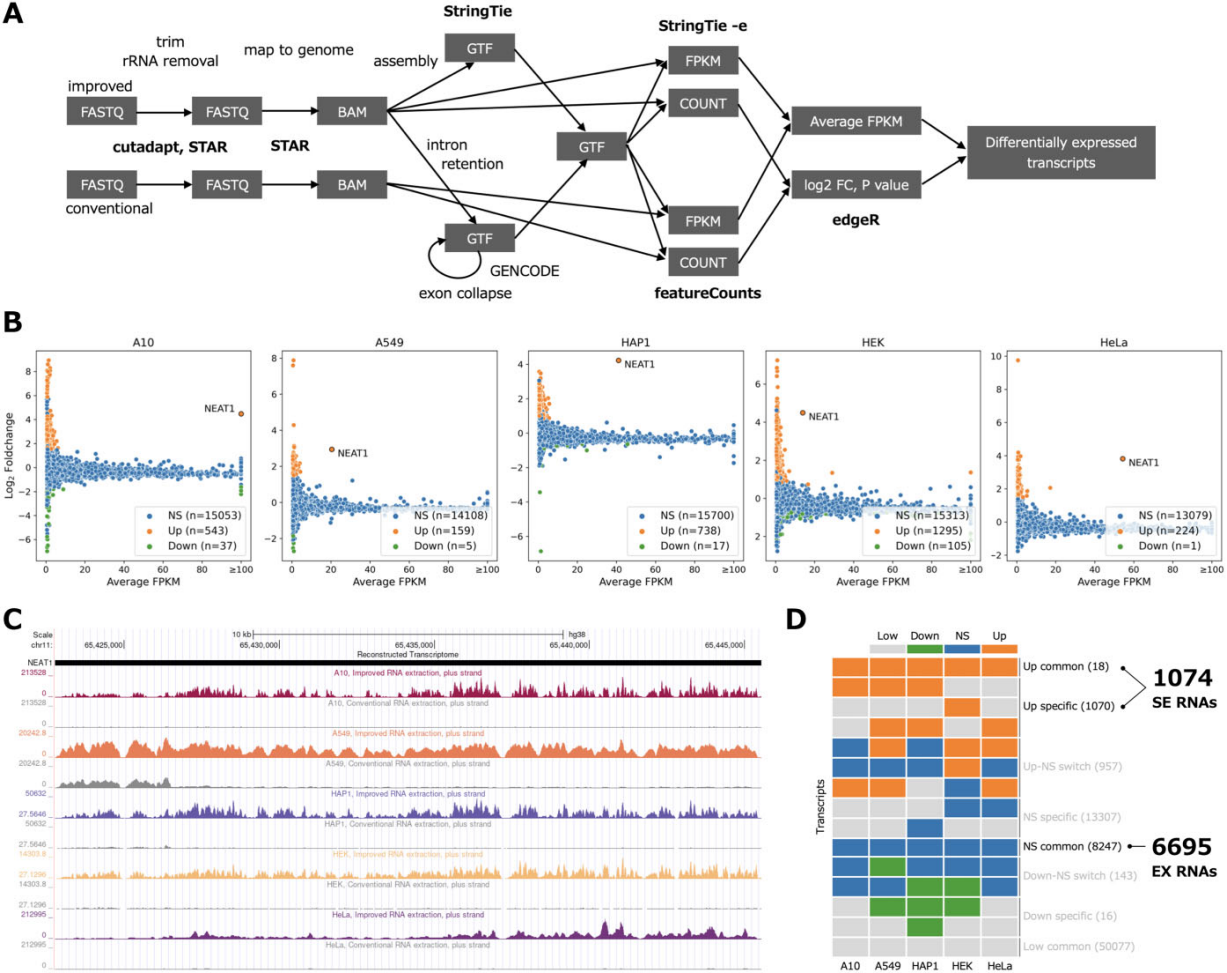
Identification of semi-extractable RNAs. (**A**) RNA-seq data analysis workflow. (**B**) Identification of semi-extractable/up-regulated RNAs (Up, orange), down-regulated RNAs (Down, green) and non-significant RNAs (NS, blue) from A10, A549, HAP1, HEK and HeLa cells. (**C**) NEAT1 was simultaneously detected as a semi-extractable RNA in the five cells. (**D**) Schematic of defining semi-extractable RNAs across five cells. A total of 1074 semi-extractable (SE) RNAs and 6695 extractable (EX) RNAs were obtained after merging from the five cells. In the case that both a representative transcript and an intron-retaining transcript are present in a gene, the former is removed. Low, low-expressed; Down, down-regulated; NS, non-significant; Up, up-regulated. See the Materials and Methods and Supplementary Data S2 for details.

To build a reference transcriptome for identification of the semi-extractable RNAs and intron retention, transcriptome reconstruction was performed based on the RNA-seq data produced by the improved RNA extraction (Figure 1A). The rationale for this approach is based on our observation that numerous semi-extractable RNAs are not properly annotated in the existing public databases. For example, hundreds of readthrough downstream-of-gene (DoG) transcripts were discovered to be semi-extractable and reported in another study (53). Semi-extractable RNAs may be the products and intermediates of various steps (e.g. transcription, processing and degradation) and thus contain intronic sequences or partially missing exonic sequences. Further, a semi-extractable RNA may not be available in the existing gene annotations, because it can be derived from intergenic regions.

The multi-mapping reads were removed prior to evaluating intron retention. Most of the RNA-seq reads in this study were short single-ended (36 nt), maintaining an empirically low unique mapping rate (71% on average, see Supplementary Table S1). Uniquely mapped reads (referred to as uniq-reads) have a higher confidence than multi-mapping reads of ambiguous origin. For single-ended reads, multi-mapping reads tended to cause higher read coverage over regions including simple repeats and/or low-complexity sequences (Supplementary Figure S1, bottom). Such ambiguous regions even confounded the surrounding high-confidence regions that were supported by uniq-reads. The above situation was not alleviated by applying longer paired-end reads (101 nt, Supplementary Figure S1, top). Accordingly, we concluded that multi-mapping reads may lead to ambiguous transcriptome construction, especially in regions containing simple repeats and/or low complexity sequences.

For each gene, all exons were collapsed to obtain a representative transcript. For the remaining intron(s), we applied the retention score (RS) to quantify its retention level based on the read coverage. The RS value reflects the expression ratio of the intron relative to its flanking exons. We combined the introns with RS values >0.1 with the original representative transcript to obtain an intron-retaining transcript. In addition, for the intergenic region, we adopted a genome-based transcriptome assembly to construct intergenic transcripts. Finally, we obtained a reference transcriptome containing 57 001 representative transcripts, 13 702 intron-retaining transcripts and 3132 intergenic transcripts (Supplementary Data S1).

### A total of 1074 semi-extractable RNAs were identified across five human cell lines

For each reference transcript, we quantified its semi-extractability using the expression increment obtained by the improved extraction method versus the conventional extraction method. A larger increment indicates higher semi-extractability of the transcript. We defined significant (*P*-value < 0.05) up-regulated transcripts as semi-extractable RNAs. Finally, 159–1295 semi-extractable RNAs were identified from each of the five cell lines (Figure 1B; Supplementary Data S3). NEAT1 lncRNA has been reported to be the most remarkable semi-extractable RNA in HeLa cells (29). This result was reproduced using HeLa cells, as shown in Figure 1B and C. NEAT1 was found to exhibit consistent semi-extractability in four other cell lines. Moreover, the expression level of NEAT1 was almost the highest among all semi-extractable RNAs.

We proceeded to determine whether transcripts other than NEAT1 exhibited stable semi-extractability across various cell lines. We investigated the overlap between the semi-extractable RNAs identified in A10, A549, HAP1, HEK and HeLa cells (Figure 1D). A total of 1074 different semi-extractable (SE) RNAs were detected in the five cell lines. Of these genes, most were specifically expressed in only some of the five cell lines and exhibited semi-extractability. Interestingly, we discovered that 18 transcripts, namely ASAP1, DANT2, EXT1, FTX, IGF1R, LIMS1, NEAT1, PHF21A, PVT1, SCMH1, STRG.3024.1, TBL1X, TCF7L2, TVP23C-CDRT4, UBE2E2, ZCCHC7, ZFAND3 and ZSWIM6, exhibited consistent and stable semi-extractability in all cell lines (Figure 1D; Supplementary Data S3). NEAT1, DANT2 and FTX are lncRNAs, STRG.3024.1 is a novel transcript identified in the intergenic region, and the remaining 14 transcripts encode proteins. For control, we identified 6695 extractable (EX) RNAs that were expressed in all five cell lines. These RNAs showed no significant difference in expression between the improved and conventional extraction methods (Figure 1D). In addition, all transcripts were prepared from the reference transcriptome as a background group (BG).

### Semi-extractable RNAs as a platform to provide RNA–RNA interactions

To investigate the distribution of semi-extractable RNAs in the chromatin, we compared their origins with the chromatin states downloaded from the ENCODE project (Table 1). Compared with extractable RNAs, semi-extractable RNAs were more enriched in repressed (polycomb repressed and low activity proximal to active states) and repetitive/heterochromatin (heterochromatin/repetitive/copy number variation) regions, with limited distribution in active promoter regions. In particular, there was a significant enrichment (SE/EX = 28.82) of semi-extractable RNAs in polycomb-repressed regions. This enrichment could suggest that these genes are under regulatory control by Polycomb proteins. One possibility is that the semi-extractable genes are involved in processes that are not essential for cell survival. For example, genes that are only expressed during early development or in specialized cells might be more likely to be repressed by Polycomb proteins.

**Table 1.** Semi-extractable RNAs are preferentially transcribed from the repressed and heterochromatin/repetitive regions

ChromHMM states	%SE	%EX	%BG	SE/EX	SE/BG
Polycomb repressed	4.85	0.20	4.41	23.82	1.10
Heterochromatin/repetitive/ copy number variation	29.22	4.07	16.80	7.18	1.74
Low activity proximal to active states	50.44	31.02	44.67	1.63	1.13
Candidate weak enhancer/DNase	3.74	3.56	4.91	1.05	0.76
Distal CTCF/candidate insulator	1.11	1.16	1.47	0.95	0.75
Candidate strong enhancer	1.15	1.32	1.39	0.88	0.83
Inactive promoter	0.07	0.18	0.26	0.41	0.28
Promoter flanking	0.42	2.15	0.94	0.19	0.44
Transcription associated	7.99	46.37	18.92	0.17	0.42
Active promoter	0.63	9.80	3.87	0.06	0.16

The chromatin state in HeLa cells was annotated in advance using ChromHMM and obtained from the ENCODE project. The percentages of various chromatin states in the transcribed regions of semi-extractable RNAs (%SE), extractable RNAs (%EX) and reference/background RNAs (%BG) were calculated separately. The ratio of %SE to %EX (SE/EX) and %BG (SE/BG) measures the transcriptional preference of semi-extractable RNAs in different chromatin states. Sorted by SE/EX column in descending order.

**Figure 2.**
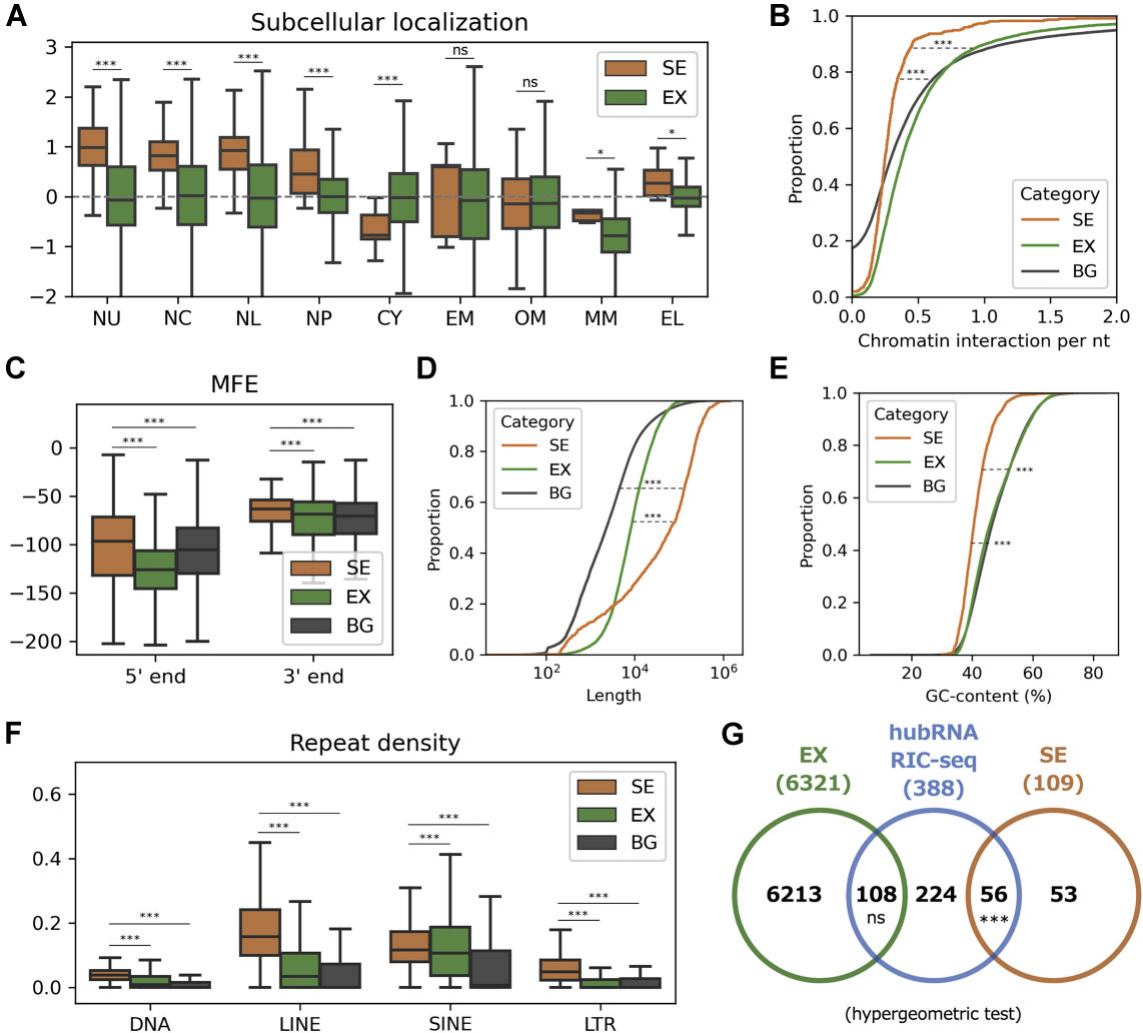
Characterization of semi-extractable RNAs. (**A**) Comparing subcellular RNA localization measured by APEX-seq fold changes in HEK cells. Increasing values indicate higher abundance in the corresponding subcellular fractions. NU, nucleolus; NC, nucleus; NL, nuclear lamina; NP, nuclear pore; CY, cytosol; EM, endoplasmic reticulum (ER) membrane; OM, outer mitochondrial membrane; MM, mitochondrial matrix; EL, ER lumen. (**B**) Chromatin–RNA interactions measured by iMARGI in HEK cells. (**C**) Comparing the minimum free energy (MFE) in the 5′ and 3′ end regions that are 300 nt in length. MFE was calculated based on RNAfold. Cumulative density function analysis of (**D**) length in nucleotide and (**E**) G and C content. (**F**) Repeat elements are significantly enriched in semi-extractable RNAs. SINE, short interspersed nuclear elements; DNA, DNA transposons; LINE, long interspersed nuclear elements; LTR, long terminal repeat. (**G**) Venn diagram analysis of semi-extractable RNAs and hub RNAs detected by RIC-seq in HeLa cells. ***P-value < 0.001, **P-value < 0.01, *P-value < 0.05; ns, non-significant (Wilcoxon rank-sum test is indicated if not otherwise specified). SE, semi-extractable RNAs; EX, extractable RNAs; BG, all background/reference RNAs.

We proceeded to examine the subcellular localization of the semi-extractable RNAs. For each transcript, we calculated the degree of preference for nine subcellular fractions from publicly available APEX-seq data (42). Semi-extractable RNAs were significantly (*P*-value < 0.001) enriched in the nuclear compartments, including the nucleolus and nuclear lamina (Figure 2A). We further investigated the association of semi-extractable RNAs with chromatin using public iMARGI data (45). Semi-extractable RNAs were found to be disassociated from chromatin (Figure 2B). Overall, semi-extractable RNAs appear to be localized in the nucleus but isolated from the chromatin.

NEAT1 forms paraspeckles through specific sequence features and an RNA-based interactome (26,28). As NEAT1 was also identified as a consistent semi-extractable RNA across cell lines in this study, we were curious as to whether semi-extractable RNAs possessed sequence characteristics similar to those of NEAT1. First, we determined whether the 5′ and 3′ ends of the semi-extractable RNAs had strong RNA structures to maintain RNA stability (28). We used the MFE of a sequence as a proxy for measuring the strength of the RNA structure. For an RNA sequence, a lower MFE indicates a higher propensity for strongly structured RNA. Surprisingly, the 5′ and 3′ ends of semi-extractable RNAs tended to have weak RNA structures (Figure 2C). In addition, we observed that the semi-extractable RNAs were significantly longer (Figure 2D) and with lower GC content (Figure 2E) than the extractable RNAs. Repeat elements (particularly LINEs) were significantly enriched in semi-extractable RNAs (Figure 2F). Based on the above observations, we hypothesized that semi-extractable RNAs are potential platforms for interactions with other RNAs. To test this hypothesis, we obtained 642 hub RNAs, which were detected to form protein-mediated RNA–RNA interactions with multiple RNAs from public RIC-seq data (46). Venn diagram analysis revealed that hub RNAs were significantly enriched (51.38%, 56 of 109) in the semi-extractable RNAs (Figure 2G). However, such enrichment of direct RNA–RNA interactions as defined in the PARIS public dataset were not observed (4.59%, 5 of 109; Supplementary Figure S2).

### Multifunctionality of the semi-extractable RNAs as reflected in clustered RBPs

**Figure 3.**
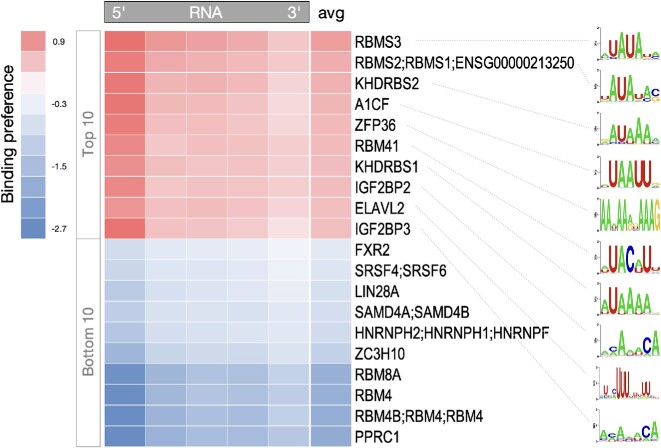
Motif enrichment analysis of semi-extractable RNAs. RBP binding preferences in different positional regions of semi-extractable RNAs, controlled with extractable RNAs. Here, the *x*- and *y*-axes represent RNAs and RBPs, respectively. RNA was divided equally into five regions, and the RBP binding density within the regions was predicted using FIMO. The avg column indicates the average binding preference of RBP over the whole RNA. The result is sorted by the avg column. RBP binding sequence motifs are shown on the right. See Supplementary Figure S3 for details.

We next explored the RBPs that bound to the semi-extractable RNAs. We downloaded the binding sequence motifs of 400 RBPs obtained by experimental validation from the CISBP-RNA database and used them to predict the binding preference of RBPs on semi-extractable RNAs. We found that RBPs that recognize AU-rich sequences were preferentially associated with semi-extractable RNAs (Figure 3; Supplementary Figure S3). AU-rich elements have been reported in the 3′-untranslated regions (UTRs) of many mRNAs and are associated with the regulation of RNA stability (54,55). Interestingly, RBPs recognizing AU-rich elements were concentrated in the 5′-terminal regions of the semi-extractable RNAs (Figure 3), implying that AU-rich elements in semi-extractable RNAs may be involved in other as yet unknown functions.

**Figure 4.**
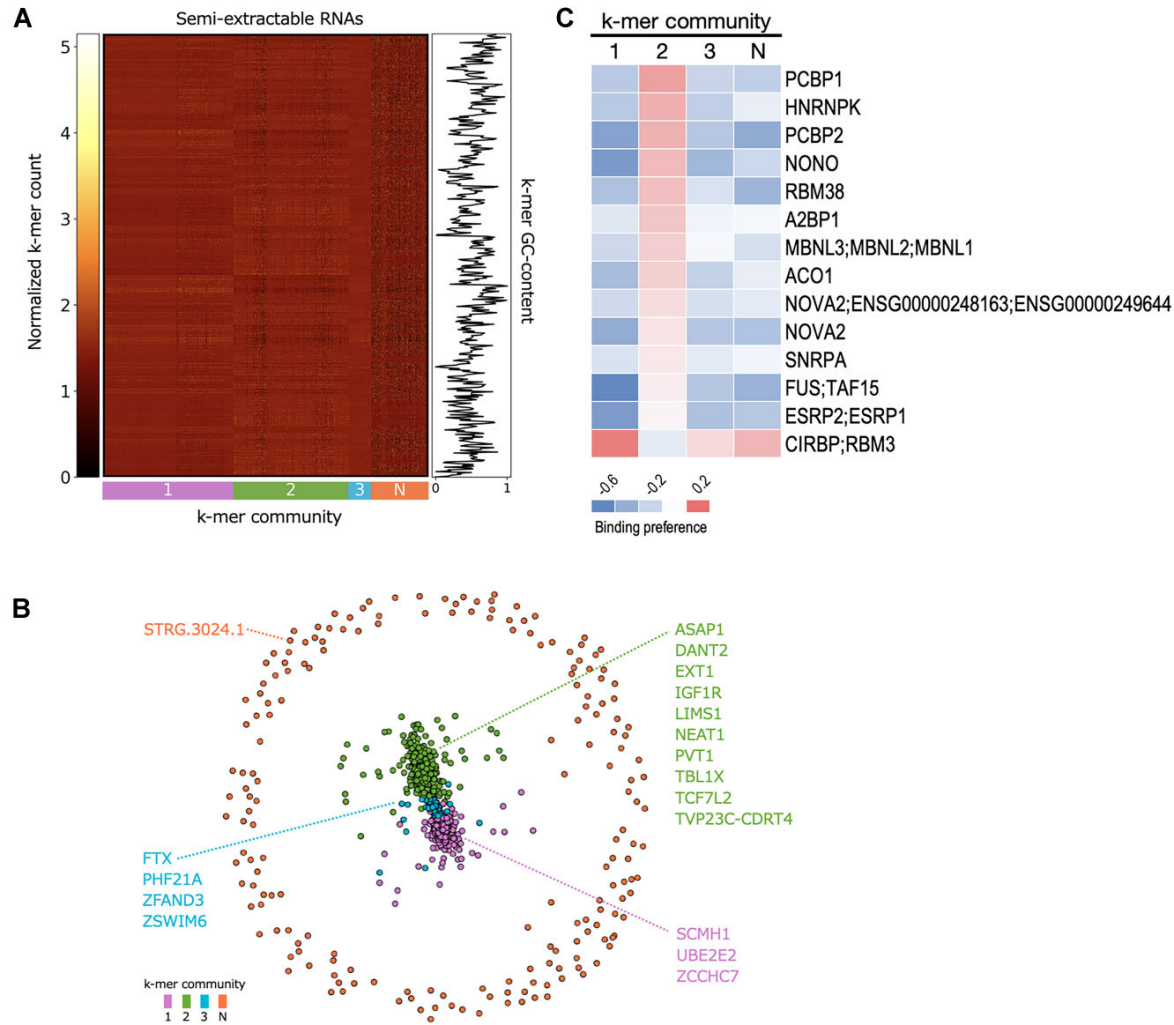
Clustering analysis of semi-extractable RNAs. (**A**) Louvain-assigned community of semi-extractable RNAs at k-mer length 6, with semi-extractable RNAs and k-mers on the *x*- and *y*-axes, respectively. Normalized k-mer count ranges from black (lowest) to yellow (highest). GC content of the k-mers is shown on the right panel. A side bar of the k-mer community is shown below the *x*-axis. N means the null community. (**B**) Network graph of semi-extractable RNAs. RNA names are colored by their Louvain community assignment. Eighteen RNAs exhibiting consistent semi-extractability in five cell lines were labeled. (**C**) RBP binding preference analysis was performed for each semi-extractable RNA community separately. Only the specific RBP binding preferences (positive and negative) for community 2 are shown. See Supplementary Figure S5 for details.

In addition, the reported paraspeckle RBPs enriched in NEAT1 (28) did not have a global binding preference for semi-extractable RNAs (Supplementary Figure S3). Hence, we hypothesized that the semi-extractable RNAs might contain functionally diverse RNAs, including a group that possesses functions similar to that of the NEAT1 constituent paraspeckles. Accordingly, we divided the semi-extractable RNAs into four potential functional groups/communities based on sequence similarity, determined by the Pearson’s correlation of k-mer profiles (Figure 4A). Among the 18 stable semi-extractable RNAs, 10 including NEAT1, PVT1 and others belonged to group 2 (Figure 4B). Furthermore, we examined the above four groups of semi-extractable RNAs for RBP binding preference. Paraspeckle RBPs, such as FUS and NONO, preferentially bound to group 2 containing NEAT1 (Figure 4C). These results are consistent with those of a previous study (28).

## DISCUSSION

In this study, 1074 semi-extractable RNAs were systematically identified from five human cell lines, thereby providing an essential resource for studying RNA-centric phase separation. Biomolecular condensates without membranes are typically formed via phase separation in cells. Most previous studies have focused on the role of various proteins in forming phase-separated structures, and many proteins associated with phase separation have been explored (1,13–15). However, numerous researchers have recently turned their attention to the role of RNA in phase separation (16–21). NEAT1 has been reported to act as an architectural RNA to form a membrane-less condensate in the nucleus, called the paraspeckle (22–25). Previous studies have experimentally verified that semi-extractable RNAs, including NEAT1, can induce the formation of nuclear bodies (29). Therefore, the RNAs contained in condensates could be poorly harvested by conventional RNA extraction and exhibited semi-extractability. Thus, the semi-extractable RNAs detected in this study may have been derived from various phase-separated condensates. Such semi-extractable RNAs may be segregated into condensates by specific biological functions. However, GO analysis showed that semi-extractable RNAs were involved in a broad range of biological processes (Supplementary Figure S4). We proposed the following two hypotheses to explain this result. First, the semi-extractable RNAs may be a mixture of RNAs derived from condensates with different biological functions. As semi-extractable RNAs can be further classified according to the type of condensates, the specific biological functions involving these RNAs could be identified. Second, semi-extractable RNAs may be involved in specific biological regulatory processes as RNA molecules, and these functions are not detectable by GO analysis based on protein function and phenotype annotation.

According to subcellular localization analysis of semi-extractable RNAs, they were enriched in the nuclear compartments. This phenomenon is consistent with the previous observation that semi-extractable RNAs are primarily derived from the nuclear bodies (29); this may be due to the dynamic exchange of contents (3), including RNAs, among the nucleolus, nucleus, nuclear lamina and nuclear pore. The semi-extractable RNAs were divided into four groups that may perform different biological functions based on sequence similarity (Figure 4B). Among them, the semi-extractable RNAs in group 2, where NEAT1 is located, preferentially bind to some known paraspeckle RBPs (i.e. NONO and FUS) (Figure 4C), implying that this group of semi-extractable RNAs may possess similar functions to NEAT1 in constituting the granule backbone. PVT1, as a stable semi-extractable RNA in this group (Supplementary Data S3), was observed to form a complex network as hub RNAs with other RNAs through RBP-mediated RNA–RNA interactions, which can form granule-like foci in the nucleus, and PVT1 foci do not intersect with known nuclear bodies (29,46). PVT1 is a neighbor of the well-known oncogene, MYC, and has been reported to be involved in the regulation of cancer development (56–61). The semi-extractable property of PVT1 implies a new aspect of phase separation for investigating its molecular regulation in the mechanism of tumorigenesis. FTX in group 3 is involved in X chromosome inactivation as a positive regulator of XIST (62). This function has been reported to depend on FTX transcription, rather than its RNA product (63). However, the semi-extractability of FTX suggests that its RNA product may be involved in X chromosome inactivation via intracellular condensates. XIST has been reported to form a phase-separated compartment by interacting with multiple RBPs (20,64,65). However, in this study, the XIST was not observed to be semi-extractable. ZCCHC7 in group 1 is involved in RNA quality regulation after translation into proteins (66), especially viral RNA degradation (67). The semi-extractability of ZCCHC7 implies that its RNA product may be harbored in the cell as biomolecular condensates, without being eagerly used for protein production, but can rapidly respond to the invasion of pathogenic RNAs.

Numerous repetitive sequences were identified in the semi-extractable RNAs (Figure 2F), which is not consistent with our speculations, as we discarded the multi-mapping reads that may result from repetitive sequences. There could be several potential reasons for these results. First, many reads may be mapped to non-repetitive regions for repeat-containing RNAs, allowing the expression levels of these RNAs to be detected. Second, reads containing repetitive sequences may still be uniquely mapped owing to mutations or unique flanking sequences in the repeats. Third, the default parameters we use in the transcript assembly cause some of the read coverage areas that are close together to be merged, which probably facilitated inclusion of repetitive sequences. Consistently, many RNAs that contain repeats have been reported to be associated with phase separation. For example, CTN-RNA was found to be distributed in mouse paraspeckles. CTN-RNA contains three inverted repeats from SINEs, which are thought to affect A-to-I editing and nuclear retention (68). CAG-repeat-containing RNA was observed to co-localize with nuclear speckles that sequester splicing factors under *in vitro* conditions (69). HSATIII lncRNAs mainly consist of primate-specific satellite III repeats, which form nuclear stress bodies under thermal stress conditions (70) and recruit specific proteins, such as heat shock factor, chromatin-remodeling complex and splicing factors (21,71,72). The middle domain of NEAT1 contains repetitive sequences from LINEs and SINEs, and this region recruits NONO dimers to trigger paraspeckle assembly (28). A systematic analysis of the potential role of repetitive sequences in the formation of RNA condensates could deepen our understanding of the biological mechanism of phase separation (73,74).

Finally, we opted to discuss possible limitations and directions for further work in this study. First, due to the various biases of short read RNA-seq (e.g. RNA fragmentation, PCR amplification and sequence context), the transcriptome may not be assembled accurately. We may consider adding nanopore direct RNA-seq data to assist in obtaining full-length reference transcripts (75). Second, we compared semi-extractable RNAs with public experimental data (e.g. iMARGI, RIC-seq and APEX-seq). Of note, these experimental data involved conventional RNA extraction and thus may have lost the information on semi-extractable RNAs. Repeating the above experiments while applying improved RNA extraction is a necessary direction of work to be completed. Fourth, various stress conditions can induce the formation of different phase-separated condensates (76–78). Therefore, exploring RNA semi-extractability under various stress conditions is expected to provide important clues for our study of the potential function of phase separation in the cellular stress response (Supplementary Data S3 and Figure S6, under analysis). Subsequent efforts will focus on RNAs that exhibit semi-extractability under specific stimulus conditions. Finally, there is growing evidence that RNA post-transcriptional modifications can regulate the dynamics of phase separation (79–81). An interesting direction of research is to investigate whether RNA modifications are associated with the semi-extractability of RNAs.

## CONCLUSION

To the best of our knowledge, this study provides the first dataset of genome-wide semi-extractable RNAs across cell lines (Supplementary Data S3). This resource is expected to guide the exploration of RNA-based phase separations. Future use of semi-extractable RNAs in conjunction with an RNA-centric interactome (45,46,82–84) will shed light on the molecular basis of the RNA-induced phase separation within cells.

## Supplementary Material

gkad567_Supplemental_File

## Data Availability

The conventional and semi-extractable RNA-seq of A10, A549, HAP1 and HEK cells have been deposited in the DDBJ Sequence Read Archive (DRA, https://www.ddbj.nig.ac.jp) under accession numbers DRA009793, DRA012807, DRA012808, DRA012810 and DRA014991. Published RNA-seq of HeLa cells was retrieved from the Gene Expression Omnibus (GEO, https://www.ncbi.nlm.nih.gov/geo) under accession number GSE80589.
