# Supplementary material for: Landscape of semi-extractable RNAs across five human cell lines
Source: Nucleic Acids Res. 2023 Jul 19;51(15):7820–31. doi: 10.1093/nar/gkad567 (PMC10450185; doi:10.1093/nar/gkad567)
Supplement: gkad567_Supplemental_File [file gkad567_supplemental_file.pdf]

Table S1. RNA-seq mapping statistics.

Sample	RNA Extraction	Treatment	Read Length	Library Layout	No. of Reads	Uniquely Mapped Reads %
A10_Conv	Conventional	Control (w/o stress treatment)	36	single-end	47280375	64.10%
A10_Impr	Improved (extensive needle shearing)	Control (w/o stress treatment)	36	single-end	60868204	72.82%
A549_Conv	Conventional	Control (w/o stress treatment)	101	paired-end	38256251	86.50%
A549_Impr	Improved (extensive needle shearing)	Control (w/o stress treatment)	101	paired-end	37322651	89.88%
HAP1_Conv	Conventional	Control (w/o stress treatment)	36	single-end	158922724	74.17%
HAP1_Impr	Improved (extensive needle shearing)	Control (w/o stress treatment)	36	single-end	146822541	77.86%
HEK_Conv	Conventional	Control (w/o stress treatment)	36	single-end	106083126	69.57%
HEK_Impr	Improved (extensive needle shearing)	Control (w/o stress treatment)	36	single-end	121287334	79.52%
HeLa_Conv	Conventional	Control (w/o stress treatment)	101	paired-end	90176187	93.41%
HeLa_Impr	Improved (extensive needle shearing)	Control (w/o stress treatment)	101	paired-end	83010947	94.02%
A10_CagA_Conv	Conventional	CagA (virulence factors) induction	36	single-end	103196273	61.80%
A10_CagA_Impr	Improved (extensive needle shearing)	CagA (virulence factors) induction	36	single-end	113259748	68.07%
HAP1_HSonly_Conv	Conventional	Heat treatment 2h	36	single-end	42487696	69.07%
HAP1_HSonly_Impr	Improved (extensive needle shearing)	Heat treatment 2h	36	single-end	50728430	75.44%
HAP1_HSrec_Conv	Conventional	Heat treatment 2h + Recovery 1h	36	single-end	124590685	73.08%
HAP1_HSrec_Impr	Improved (extensive needle shearing)	Heat treatment 2h + Recovery 1h	36	single-end	158830301	80.58%
HAP1_LowO2_Conv	Conventional	Hypoxia treatment	36	single-end	38039571	63.86%
HAP1_LowO2_Impr	Improved (extensive needle shearing)	Hypoxia treatment	36	single-end	50454927	74.86%
HAP1_MG132_Conv	Conventional	MG132 (proteosome inhibitor) treatment	36	single-end	43066243	64.07%
HAP1_MG132_Impr	Improved (extensive needle shearing)	MG132 (proteosome inhibitor) treatment	36	single-end	33243084	69.60%
HAP1_NoSrm_Conv	Conventional	Serum starvation treatment	36	single-end	41545470	60.58%
HAP1_NoSrm_Impr	Improved (extensive needle shearing)	Serum starvation treatment	36	single-end	41213572	69.63%
HEK_Sorbi_Conv	Conventional	Osmotic pressue treatment	36	single-end	151011252	66.06%
HEK_Sorbi_Impr	Improved (extensive needle shearing)	Osmotic pressue treatment	36	single-end	165714234	79.48%

Figure S1. Multi-mapping reads lead to ambiguous coverage in intronic region. As an example of the DANT2 locus, either paired-end (PE, 101 nt) or single-end reads (SE, 36 nt), multiple mapped reads (multi) resulted in a read coverage of simple repeat/low complexity (dashed box) in intronic region. Read mapping were performed with STAR. Impr: improved RNA extraction, Conv: conventional RNA extraction.

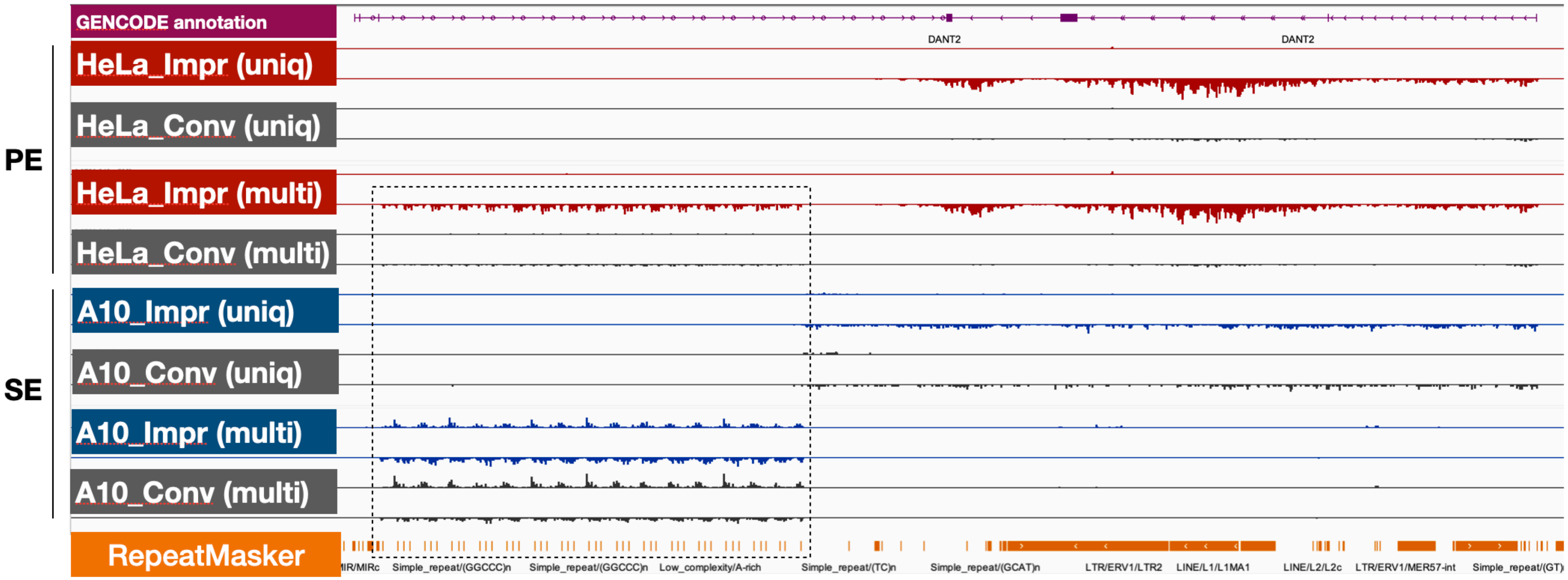

Figure S2. Venn diagram analysis of semi-extractable RNAs and hub RNAs detected by PARIS in HeLa cell. ***: p-value < 0.001, **: p-value < 0.01, *: p-value < 0.05, ns: no significance. SE: semi-extractable RNAs, EX: extractable RNAs.

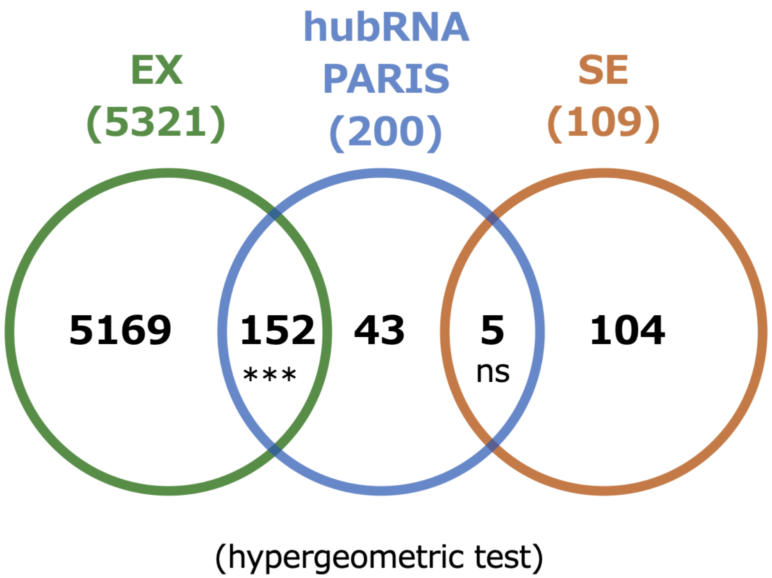

Figure S3. RBP binding preferences of semi-extractable RNAs.

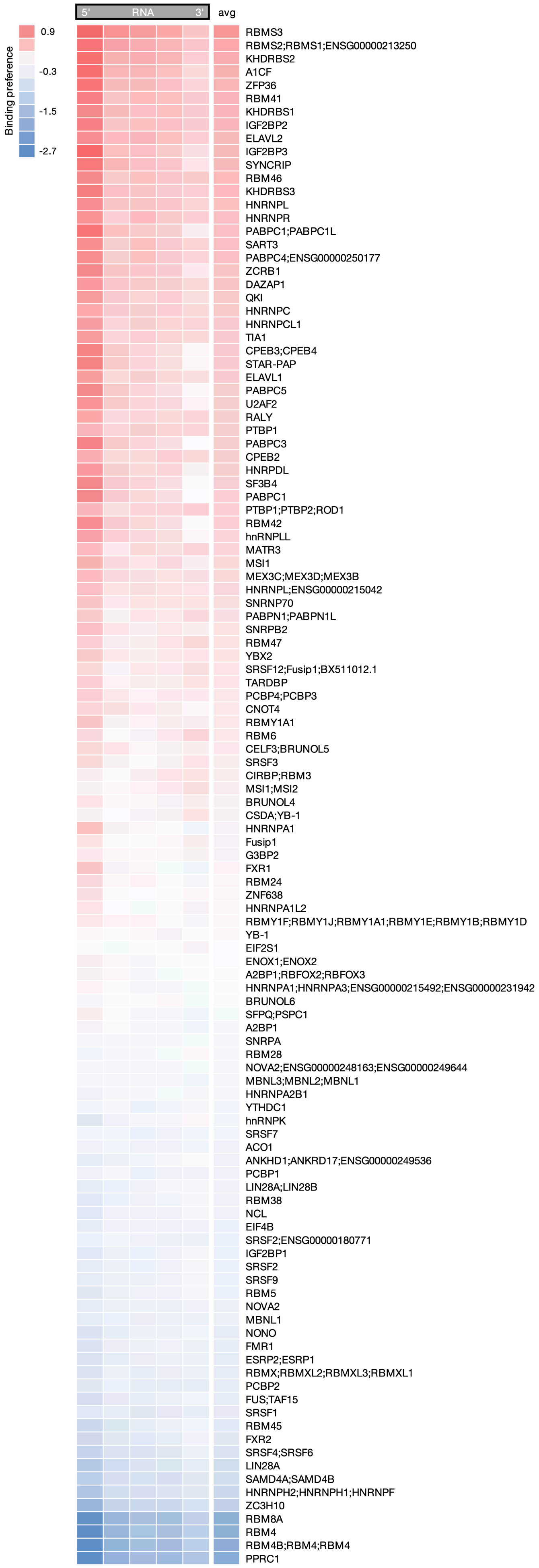

Figure S4. GO analysis for semi-extractable genes. GO analysis was conducted for 714 semi-extractable genes with g:Profiler. MF for molecular function, BP for biological process, and CC for cellular component.

source	term_name	term_id	negative_log10_of_adjusted_p_value
MF	protein binding	GO:0005515	4.522
MF	metal ion binding	GO:0046872	3.356
MF	cation binding	GO:0043169	3.050
BP	system development	GO:0048731	16.765
BP	multicellular organism development	GO:0007275	16.561
BP	nervous system development	GO:0007399	16.100
BP	anatomical structure development	GO:0048856	12.925
BP	generation of neurons	GO:0048699	11.841
BP	neuron differentiation	GO:0030182	11.552
BP	neuron development	GO:0048666	10.123
BP	regulation of cellular process	GO:0050794	10.107
BP	developmental process	GO:0032502	9.907
BP	neurogenesis	GO:0022008	9.504
BP	biological regulation	GO:0065007	8.549
BP	anatomical structure morphogenesis	GO:0009653	8.185
BP	regulation of biological process	GO:0050789	8.095
BP	animal organ development	GO:0048513	7.395
BP	neuron projection development	GO:0031175	7.240
BP	cell morphogenesis involved in neuron differentiation	GO:0048667	6.935
BP	cell junction organization	GO:0034330	6.083
BP	cell morphogenesis involved in differentiation	GO:0000904	5.987
BP	cell part morphogenesis	GO:0032990	5.958
BP	plasma membrane bounded cell projection morphogenesis	GO:0120039	5.909
BP	cell projection morphogenesis	GO:0048858	5.813
BP	neuron projection morphogenesis	GO:0048812	5.688
BP	cellular component morphogenesis	GO:0032989	5.595
BP	cell projection organization	GO:0030030	5.328
BP	plasma membrane bounded cell projection organization	GO:0120036	4.990
BP	synapse organization	GO:0050808	4.921
BP	negative regulation of cellular process	GO:0048523	4.772
BP	axonogenesis	GO:0007409	4.660
BP	cell development	GO:0048468	4.655
BP	proteoglycan biosynthetic process	GO:0030166	4.654
BP	regulation of neuron projection development	GO:0010975	4.334
BP	cell morphogenesis	GO:0000902	4.197
BP	animal organ morphogenesis	GO:0009887	4.159
BP	regulation of cell projection organization	GO:0031344	4.049
BP	glycoprotein biosynthetic process	GO:0009101	3.984
BP	regulation of plasma membrane bounded cell projection organization	GO:0120035	3.798
BP	axon development	GO:0061564	3.657
BP	central nervous system development	GO:0007417	3.552
BP	negative regulation of biological process	GO:0048519	3.419
BP	cell communication	GO:0007154	3.391
BP	macromolecule biosynthetic process	GO:0009059	3.321
BP	multicellular organismal process	GO:0032501	3.293
BP	signaling	GO:0023052	3.272
BP	cellular developmental process	GO:0048869	3.166
BP	heparan sulfate proteoglycan biosynthetic process	GO:0015012	3.077
BP	cell differentiation	GO:0030154	3.073
BP	intracellular signal transduction	GO:0035556	3.009
CC	synapse	GO:0045202	7.562
CC	cell junction	GO:0030054	6.475
CC	cytoplasm	GO:0005737	5.871
CC	postsynaptic density	GO:0014069	3.292
CC	postsynapse	GO:0098794	3.180
CC	glutamatergic synapse	GO:0098978	3.055
CC	neuron projection	GO:0043005	3.021
CC	asymmetric synapse	GO:0032279	3.004

Figure S5. RBP binding preferences for semi-extractable RNA groups.

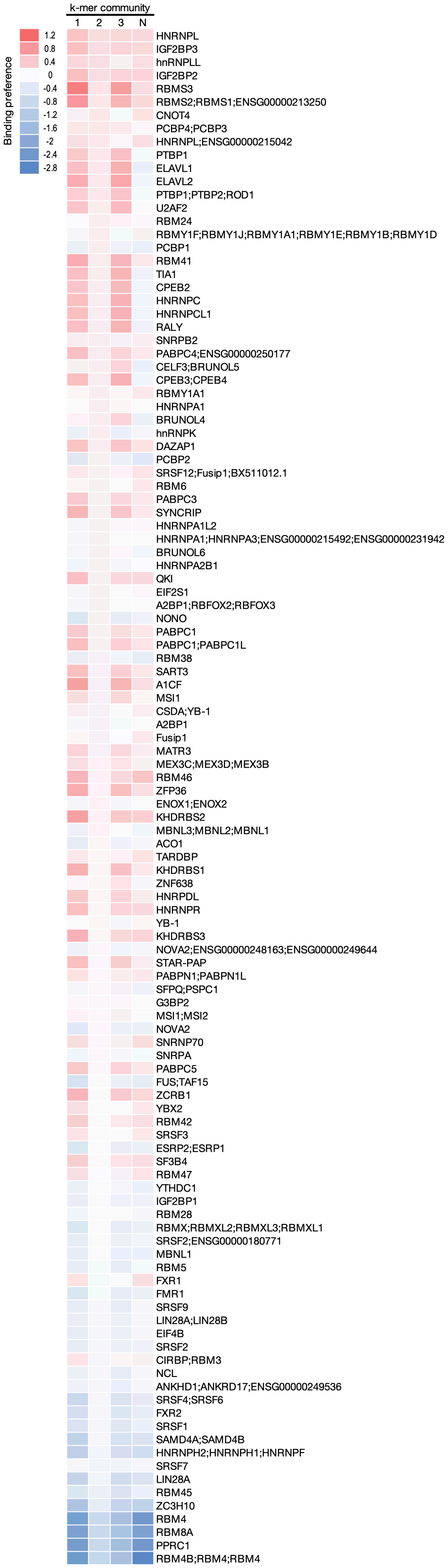

Figure S6. Semi-extractable RNAs under different stress conditions. Semi-extractable/up-regulated RNAs (Up, orange) and down-regulated RNAs (Down, green) were identified under seven stress conditions, respectively. CagA: CagA (virulence factors) induction. HSonly: heat treatment 2 hours. HSrec: heat treatment 2 hours and recovery 1 hour. LowO2: hypoxia treatment. MG132: MG132 (proteasome inhibitor) treatment. NoSrm: serum starvation treatment. Sorbi: osmotic pressure treatment.

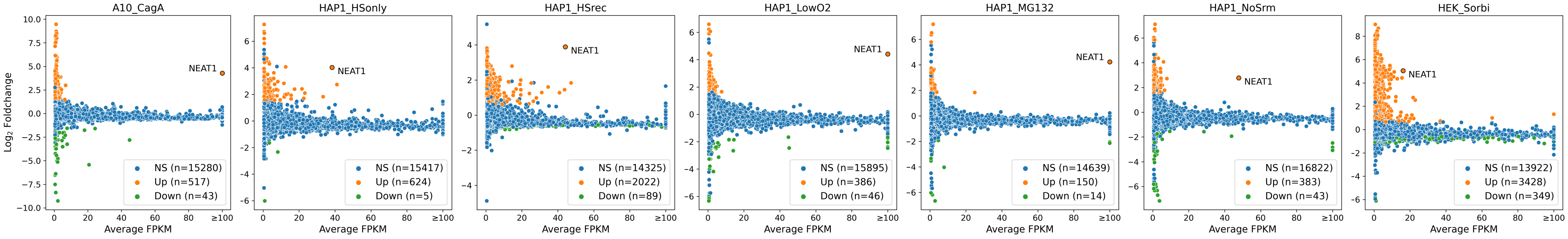

Data S1. Gene annotation file for the reference transcriptome used to identify semi-extractable RNAs. A GTF (General Transfer Format) format file containing 7,001 representative transcripts, 13,702 intron-containing transcripts (transcript names with "_RI" suffix) and 3,132 intergenic transcripts (transcript names with "STRG." prefix) obtained in this study.

Online resource: https://doi.org/10.6084/m9.figshare.22817327

Data S2. Full list of semi-extractable (SE) RNAs and extractable (EX) RNAs.

Online resource: https://doi.org/10.6084/m9.figshare.22986668

Data S3. Differential gene expression analysis across cell lines and stress conditions.

Online resource: https://doi.org/10.6084/m9.figshare.22986068
